# Differential investment in visual and olfactory brain areas reflects behavioural choices in hawk moths

**DOI:** 10.1038/srep26041

**Published:** 2016-05-17

**Authors:** Anna Stöckl, Stanley Heinze, Alice Charalabidis, Basil el Jundi, Eric Warrant, Almut Kelber

**Affiliations:** 1 Department of Biology, University of Lund, Sölvegatan 35, S-22362 Lund, Sweden

## Abstract

Nervous tissue is one of the most metabolically expensive animal tissues, thus evolutionary investments that result in enlarged brain regions should also result in improved behavioural performance. Indeed, large-scale comparative studies in vertebrates and invertebrates have successfully linked differences in brain anatomy to differences in ecology and behaviour, but their precision can be limited by the detail of the anatomical measurements, or by only measuring behaviour indirectly. Therefore, detailed case studies are valuable complements to these investigations, and have provided important evidence linking brain structure to function in a range of higher-order behavioural traits, such as foraging experience or aggressive behaviour. Here, we show that differences in the size of both lower and higher-order sensory brain areas reflect differences in the relative importance of these senses in the foraging choices of hawk moths, as suggested by previous anatomical work in Lepidopterans. To this end we combined anatomical and behavioural quantifications of the relative importance of vision and olfaction in two closely related hawk moth species. We conclude that differences in sensory brain volume in these hawk moths can indeed be interpreted as differences in the importance of these senses for the animal’s behaviour.

One central question in neurobiology is how the structure of the brain reflects its function. Since the central nervous system is one of the most energetically expensive tissues, its size is limited by production and maintenance costs^1^,^2^,^3^,^4^. Thus, in order to convey a selective advantage, increased investment leading to a larger brain (or brain areas) should improve the performance of its encoded functions^2^ and shape the behaviour of the animal^5^. This link between structure and function builds the basis for anatomical comparisons of brain regions between species and functional conclusions based on these results.

Numerous studies have quantified brain volume within and across species in insects^5^,^6^,^7^,^8^,^9^,^10^,^11^,^12^ and vertebrates (for a review^13^), and have interpreted the results in terms of functional and behavioural relevance. Comparative studies in invertebrates have successfully linked differences in brain anatomy to differences in ecology and behaviour^5^,^6^,^8^,^11^,^14^,^15^,^16^,^17^.

Routinely, large-scale comparative studies are employed for these investigations. While they have greater statistical power, the anatomical and behavioural detail for each individual species is limited for practical reasons. They can lack precision in their analyses of the behavioural relevance of size differences in brain anatomy – because they only rely on indirect measures of behaviour, or focus on few brain areas and/or total brain volume^13^. Therefore, detailed case studies are valuable complements to large scale comparative studies, and these have provided important evidence linking brain structure to function in both vertebrates^18^,^19^,^20^ and invertebrates^14^,^21^,^22^,^23^,^24^.

While invertebrate studies linking brain anatomy and behaviour have focused on aspects of learning^21^,^22^, as well as higher-order behavioural traits, such as dominance and aggressive behaviour^23^,^24^, foraging experience^21^ and task specificity^14^, here we have investigated whether differences in the size of sensory brain areas reflect differences in the relative importance of these senses in an animal’s behaviour, as suggested by anatomical work and ecological factors in Lepidopterans^5^. To this end, we quantified the relative weights of vision and olfaction in the brain morphology and behaviour of two closely related hawk moth species (*Sphingidae; Macroglossinae; Macroglossini*^25^).

We compared the diurnal hummingbird hawk moth *Macroglossum stellatarum* and the nocturnal elephant hawk moth *Deilephila elpenor*, which possess similar eye designs (superposition eyes), flight dynamics (hovering flight during foraging) and foraging behaviour. Their energetically costly hovering flight when foraging nectar from flowers^26^ forces them to make efficient and precise decisions about their food sources^27^. Previous work has already shown that naïve individuals of the diurnal species show selective preferences for food sources based primarily on visual cues, while the nocturnal species primarily prefers olfactory cues^28^, despite both species being able to learn both olfactory and colour cues^29^,^30^. Here we show that this sensory difference is present in the volume of the major visual and olfactory neuropils in lower and higher-order brain areas of the two species. Thus, differential investment in brain volume reflects the innate behavioural preferences. Furthermore, we used experienced animals to test the relative importance of visual and olfactory cues for flower choice in a quantitative behavioural learning assay. We show that the relative weights the two species give to vision and olfaction reflects the differential investment in the two sensory modalities in their brains.

## Results and Discussion

### Relative investment in vision and olfaction in lower-order sensory neuropils

**Figure 1:**
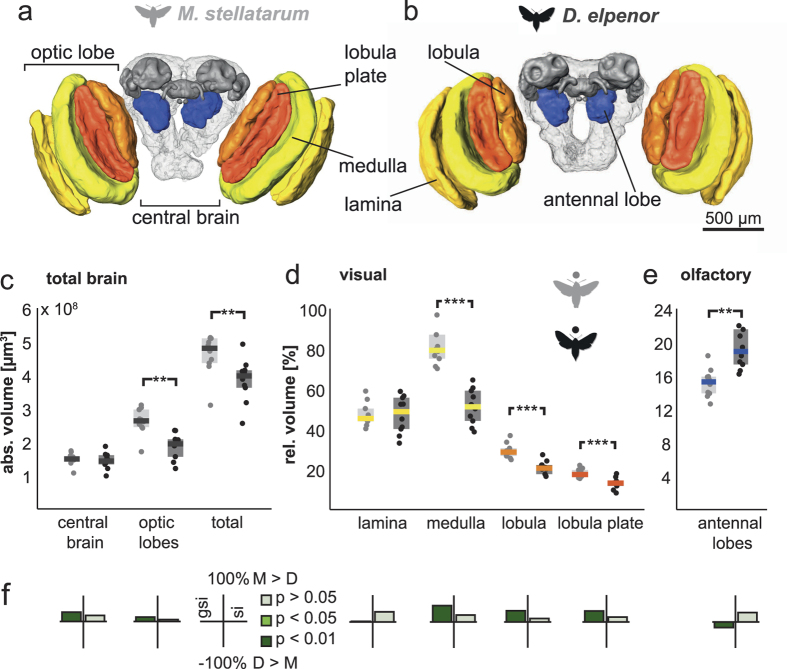
Lower-order visual and olfactory neuropils in *M. stellatarum* and *D. elpenor.* Posterior view of 3D reconstructed neuropils of the diurnal (**a**) and nocturnal (**b**) species. Lower-order structures in colour, higher-order neuropils in grey, remaining central brain neuropil in light grey. Absolute (**c**) and relative (**d**,**e**) neuropil volumes of the total brain (**c**) and visual (**d**) and olfactory (**e**) structures, respectively. Grey (diurnal) and black (nocturnal) circles show individual measurements, shaded areas interquartile ranges and horizontal bars medians (colour coded as in **a**,**b**). Significance tests with Mann-Whitney-U test: *p < 0.05, **p < 0.01, ***p < 0.001. (**f**) The grade shift index (**gsi**) illustrates scaling differences in neuropil volume between species (positive values: *M. stellatarum* > *D.elpenor*, negative values: vice versa), while the slope ratio (**si**) shows a divergence of the respective neuropil from isometric scaling with respect to the central brain. Significance is indicated by the colour. For full results and statistics see Supplementary Tables S1, S2.

Both species showed a similar gross brain layout with bilaterally symmetric optic lobes and a central brain (Fig. 1A,B). In absolute terms, the total brain neuropil volume was 20% larger in the diurnal species than in the nocturnal species (n = 10 for both species, Mann-Whitney-U-test, p = 0.009, Fig. 1C, results and full statistics for all neuropils in Supplementary Table S1), a difference entirely attributable to the optic lobes, containing the lower-order visual processing areas. The central brain neuropil volume did not differ between species. For comparing neuropil volume between species, we normalized the neuropils by the volume of the central brain (excluding the segmented sensory neuropil) in each species. We also confirmed that differences in percentage volume were not a bi-product of allometric scaling with overall brain size by identifying non-allometric grade-shifts between the neuropils of interest and total brain size (as exemplified in^17^). Full statistics for regression analysis on all neuropils are found in Supplementary Table S2. P-values in the text refer to Mann-Whitney-U-tests of neuropil volume differences between species, if not stated otherwise.

The first neuropil in the optic lobes (lamina, Fig. 1D) showed no significant volume difference between species, while the following three optic lobe neuropils – the medulla, lobula and lobula plate – were individually significantly larger (p < 0.01, U_10,10_ = 4; p<0.001, U_10,10_ = 4; p < 0.01, U_10,10_ = 7 respectively), and together nearly one third larger (p < 0.01, U_10,10_ = 7), in the diurnal species. All optic lobe neuropils scaled isometrically with the central brain in both species, and the difference in volume was purely caused by a grade shift between species (Fig. 1F). Our results are in agreement with previous studies on Lepidoptera with contrasting sensory ecologies, which showed that interspecific differences in brain composition affect the medulla and lobula more than the lamina^5^,^9^.

The ommatidia are the optical building blocks of the compound eye, each of which processes information from a single “pixel” of the visual image. *M. stellatarum* has only half as many ommatidia as *D. elpenor* (because it has a smaller eye and smaller body size)^31^, and thus its larger optic lobe volume implies that the amount of neural tissue devoted to each “pixel” is considerably larger in the diurnal than in the nocturnal species^31^,^32^.

In contrast, the lower-order olfactory processing centres, the antennal lobes, were larger in the nocturnal species (p < 0.01, U_10,10_ = 6; Fig. 1E), resulting from a grade shift between species (Fig. 1F). This difference was based on a difference in the volume of the individual processing units of the antennal lobes, the glomeruli: subtracting the central fibrous neuropil that together with the glomeruli comprises the antennal lobes gave the same result (“AL glomeruli”, Supplementary Fig. S1 and Supplementary Table S2). Both species had similar numbers of glomeruli (*M. stellatarum*: 77 (female), 77 (male); *D. elpenor*: 77 (female), 76 (male)) – thus, on average, glomeruli were also bigger in the nocturnal than the diurnal species. This difference in antennal lobe size between species was not caused by a sexual dimorphism. In most moths, males have specialized glomeruli to detect female pheromones (macroglomeruli), which can significantly exceed their female equivalents in size, and thus result in a sexual dimorphism in antennal lobe size, as demonstrated for example in the hawk moth *Manduca sexta*^7^. In *M. stellatarum*, there was no such sexual dimorphism (despite the presence of macroglomeruli, p = 0.55, U_5,5_ = 12), while there was a strong, though non-significant trend towards sexual dimorphism in *D. elpenor* (p = 0.05, U_5,5_ = 3). We tested for grade shifts between species using the antennal lobe neuropil with the volume of the macroglomeruli subtracted. The grade shift between species was still highly significant (p < 0.01, Wald test for common elevation), showing similar scaling for the non-sexual parts of the antennal lobes as for total antennal lobe volume (“AL – macroglomeruli”, Supplementary Fig. S1 and Supplementary Table S2), and thus ruling out that sexual dimorphism caused the relative volume difference between species.

Taken together, while the lower-order visual neuropils were larger in the diurnal species, the corresponding olfactory neuropil was larger in the nocturnal species, in line with previous findings in vertebrates^33^,^34^,^35^ and invertebrates^5^,^11^, underlining a fundamental trend across animal phyla to invest more strongly in vision when diurnal, and more strongly in olfaction when nocturnal.

In both sensory modalities, the differential investment in neuropil volume was manifested as enlarged individual processing units, suggesting that investment in lower-order areas did not result in a higher spatial resolution in vision or a greater dimensionality of odour space, but in a greater investment in the size of brain regions processing each type of sensory information. In the olfactory system potential advantages from dedicating more processing power to each individual glomerulus could be increased sensitivity for individual odour components, as summation over more olfactory receptor neurons would lead to bigger olfactory glomeruli. In the visual pathway equivalent investments could increase precision of colour or brightness discrimination by adding neurons that process information from each “pixel”. Moreover, larger neurons (that take up more volume) could result in faster processing through faster dendritic and synaptic information transfer^2^,^36^.

### Relative investment in vision and olfaction in higher-order brain neuropils

After finding a clear difference between visual and olfactory investment in lower-order sensory neuropils, we went on to investigate unimodal and multimodal higher-order neuropils.

**Figure 2:**
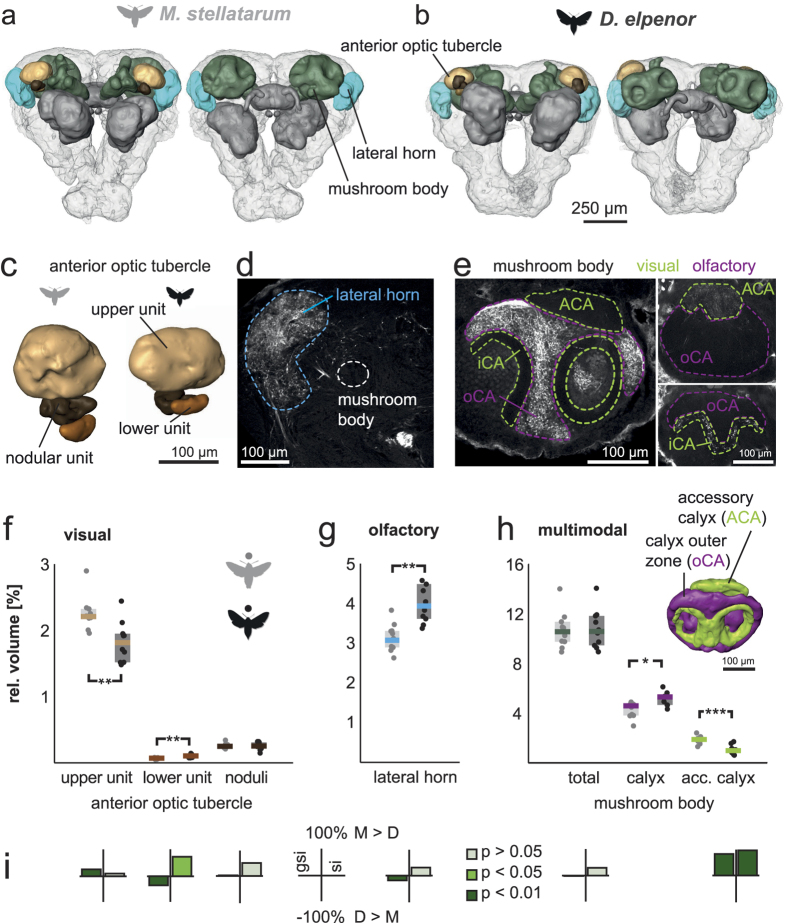
Higher-order visual and olfactory neuropils in *M. stellatarum* and *D. elpenor.* Anterior (left) and posterior (right) view of 3D reconstructed central neuropils of the diurnal (**a**) and nocturnal (**b**) species. Higher-order sensory structures in colour, other neuropils in grey, remaining neuropil in light grey (**c)**. Close-up of the anterior optic tubercle (visual). (**d**,**e)** Olfactory projections from the antennal lobes identified the lateral horn (**d**, blue line), and the olfactory input regions to the mushroom body calyx (calyx inner zone, (**e**). Lobula injections revealed visual input regions in the mushroom body calyx (**e**). Scale bars in (**c–e)** 100 μm. (**f–h).** Relative neuropil volumes of higher-order visual (**f**), olfactory (**g**) and multimodal (**h**) brain areas: grey (diurnal) and black (nocturnal) circles show individual measurements, shaded areas interquartile ranges and horizontal bars (colour code as in **a**,**b**) medians. Significance tests with Mann-Whitney-U test: *p < 0.05, **p < 0.01, ***p < 0.001. (**i**) The grade shift index (**gsi**) and the slope ratio (**si**). Colour as in Fig. 1. For full results and statistics see Supplementary Tables S1, S2.

The only clearly delineated structure in the central brain that exclusively receives visual input is the anterior optic tubercle^37^. In honeybees, its largest compartment, the upper unit, processes chromatic information^38^, which is relevant to flower choice. The upper unit was 26% larger in the diurnal moth species (p < 0.01, U_10,10_ = 11; Fig. 2F), and this difference arose from a grade shift between species. Interestingly, the lower unit, a relay centre for skylight compass information in many insects^39^,^40^,^41^, showed the reverse effect: its size was 33% larger in the nocturnal species (p < 0.01, U_10,10_ = 8). We found no significant difference between species in the volume of the functionally undescribed nodular unit (p = 0.97, U_10,10_ = 49), which scaled isometrically with the central brain volume in both species.

Analogous to the visual anterior optic tubercle, the lateral horn is a higher-order olfactory processing area that exclusively receives input from the antennal lobe^42^,^43^. As the boundaries of this brain region are not easily identified by neuropil staining, its location and extent were defined by the arborisation fields of antennal lobe projection neurons (Fig. 2D). We found the relative volume of the lateral horn to be significantly larger in the nocturnal species (p < 0.01, U_10,10_ = 9; Fig. 2G), resulting from a true grade shift between species (Fig. 2I).

Thus, the relative investment in lower-order visual and olfactory neuropils in these two hawk moth species was mirrored in the higher-order processing areas that exclusively receive either visual or olfactory input. Would this investment strategy also extend to multi-sensory brain areas? To answer this question, we investigated the mushroom bodies, which are responsible for many learning and memory processes in insects^44^,^45^ and in many species possess clearly delineated visual and olfactory input regions^46^,^47^,^48^. By tracing visual and olfactory neurons to distinct sub-compartments of the mushroom body calyx, we confirmed this segregation of sensory modalities in hawk moths (Fig. 2E), and revealed similarities to the Swallowtail butterfly^47^: the accessory calyx, as well as the calyx outer zone received visual inputs, while the calyx inner zone received olfactory innervation.

The total mushroom body volume did not differ significantly between the two species (p = 0.62, U_10,10_ = 43, Fig. 2H), and scaled isometrically with total central brain volume (Fig. 2I), demonstrating that both species invest equally in this learning and memory centre. However, the accessory calyx, associated with vision, showed a clear grade shift between species, which resulted in an 80% bigger accessory calyx volume in the diurnal species (Fig. 2H,I). There was no significant difference in calyx volume between the species. Since the calyx scaled differently with central brain volume in the two species (Supplementary Fig. 1 and Table 2), testing for a grade shift was not meaningful. Nevertheless, reconstructing the proportion of areas receiving olfactory or visual information showed that this ratio was distinctly higher in the nocturnal species (*M. stellatarum*: 76.9% +− 1.42% s.d. (n = 4), *D. elpenor*: 85.4 +− 2.9% s.d. (n = 4), p = 0.03, U_4,4_ = 0), suggesting they use a larger proportion of their calyces to process olfactory information than the diurnal species. Thus, even in this higher-order multisensory brain structure we found clear differences in sensory investment, although the overall size of the region was conserved.

Previous comparative studies on differential investment in higher-order sensory areas have focused on the multimodal mushroom bodies and revealed contrasting results; some found evidence for differential scaling of visual and olfactory regions^8^, while others did not^8^,^11^. Similarly, studies showing that higher brain structures in vertebrates do not reflect differential investment^35^,^49^ have recently been challenged by new results^34^. This has stimulated the debate whether higher processing brain regions in animals are subject to differential investment into specific brain regions (mosaic brain evolution) or concerted investment into all brain regions together (concerted brain evolution). Our results in hawk moths suggest that at least in this insect group, differential investment takes place from lower-order structures to both unimodal and multimodal higher-order structures.

### The relative importance of visual and olfactory cues in a learned foraging task

If the difference in neuropil volume between modalities results from differential evolutionary investment in each species, we would expect a corresponding difference in the behaviour that is under selective pressure^2^,^5^. Previous data on the naïve choices of foraging hawk moths for visual or olfactory cues have revealed that diurnal moths preferentially rely on colour cues while nocturnal moths prefer odour cues^28^. Combining these findings with our neuropil data shows a strong connection between differential investment into sensory brain areas and behavioural choices. Here we extended these studies and asked whether the anatomical differences are also reflected in the weight that experienced moths give to vision and olfaction when forced to make a choice between learned visual and olfactory cues.

**Figure 3:**
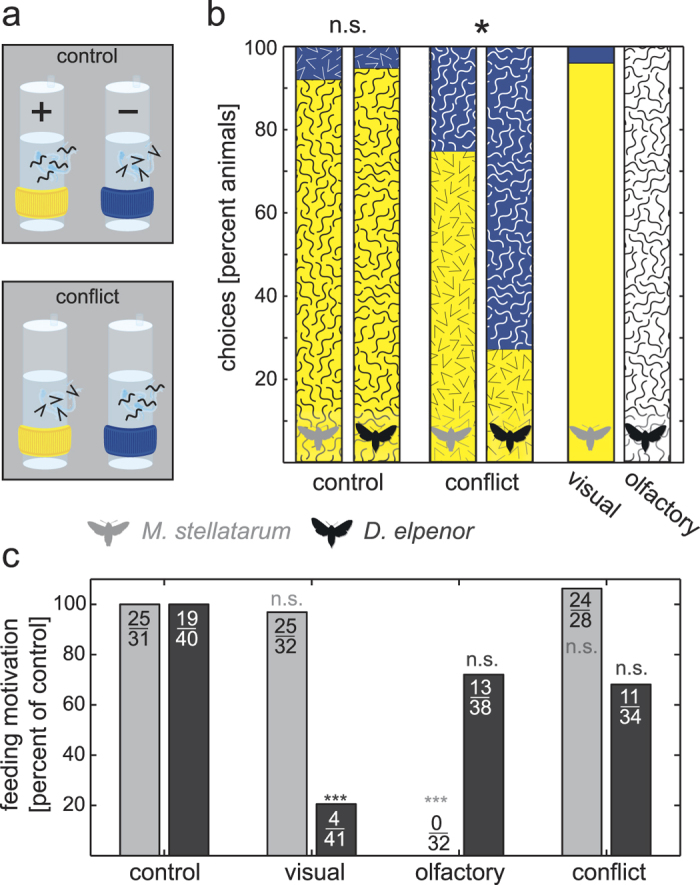
The relative importance of visual and odour cues in a foraging task. **(a)** Animals were trained to a rewarded combination of visual and olfactory cues (yellow and honeysuckle odour (waves)) and the unrewarded (blue and bergamot odour (arrows)) and tested with this combination (*control*) or a *conflict* between cues. (**b)** Choices (touching of the feeders with the proboscis, percentage of animals) when presented with the *control* and *conflict* condition, *visual* cues only in *M. stellatarum,* and *olfactory* cues only in *D. elpenor*. (**c)** Feeding motivation of moths scored as touching of the feeder with the proboscis out of all animals flying, normalized to the *control* condition for each species. Significance tests in (**b**) and (**c**), Fisher’s exact test: *p < 0.05, **p < 0.01, ***p < 0.001. For full results and statistics see Supplementary Table S3.

The moths were trained to associate the combination of a visual and an olfactory cue (yellow colour and honeysuckle odour) with a food reward, and another combination (blue colour and bergamot odour) with the absence of a reward (Fig. 3A). Moths of both species learned to choose the rewarded combination in more than 90% of choices (Fig. 3B, *control,* see also Supplementary Table S3 for all choices, and Supplementary Table S4 for full statistics). To test which sensory modality each species weights more strongly, the trained moths were presented with a conflicting combination of the visual and olfactory stimuli (the rewarded visual cue combined with the unrewarded odour and *vice versa*). While 75% of the diurnal species chose the rewarded colour over the rewarded odour, in the nocturnal species this ratio was reversed: only 27% of individuals chose the rewarded colour, while 73% chose the rewarded odour (Fig. 3B, *conflict*). This result was significant (p = 0.012, odds ratio = 0.125, Fisher’s exact test), and was in agreement with previous findings on innate choices in naïve individuals^28^.

The animal’s choice in the *conflict* situation was influenced by both sensory modalities: only 75% of the diurnal species chose vision over olfaction when in *conflict*, while 96% chose the correct colour when *only* colours were presented (Fig. 3B *visual*; p = 0.05, odd ratio = 0.125). Similarly for the nocturnal moths, 73% of the nocturnal species chose olfaction over vision, while 100% chose correctly when *only* odours were presented (Fig. 3B, *olfactory,* p = 0.08, odds ratio = 0). If the animals had disregarded the non-preferred modality, both situations would be equivalent and choice rates should have been identical.

Underlining these sensory preferences, the motivation to initiate feeding (probing the feeders with their proboscis) depended strongly on the presented sensory cues. While there was no difference in the proportion of moths probing between conditions with *both* visual *and* olfactory cues (*control* and *conflict, M. stellatarum:* p = 0.73, odds ratio = 1.44, *D. elpenor:* p = 0.24, odds ratio = 0.52), the two species showed a strong divergence when *only* visual *or* olfactory cues were presented. None of the diurnal moths initiated feeding when *only* odours were present, but behaved similarly to the *control* condition when only colours were present (p = 1.00, odds ratio = 0.86, Fig. 3C). The reverse was true for the nocturnal moths – a significantly reduced proportion initiated feeding with *only* colours present (p < 0.01, odds ratio = 0.12), whereas they showed no difference to the *control* with only odours (p = 0.26, odd ratio = 0.57) (Fig. 3C).

Thus, hawk moths learned and used both visual and olfactory cues, but when forced to make a choice between modalities, the diurnal species relied more strongly on vision, and the nocturnal species on olfaction. Taken together, the differences in investment in visual and olfactory neuropils were mirrored in the behavioural choices of trained moths.

### Neuropil volume reflects the importance of sensory information

When we combine our anatomical and behavioural results, it becomes apparent that differences in the weights that the two hawk moth species assign to visual and olfactory cues in a behavioural task are mirrored by differences in their sensory neuropil volumes. An increased visual neuropil volume is associated with a stronger reliance of visual foraging cues, while an increased olfactory neuropil volume is associated with a stronger weighting of olfactory cues. We propose that the cause for this relation is differential selection on the two sensory modalities. Increased evolutionary investment in one sensory modality, manifested as enlarged lower and higher-order neuropil volumes, should result in better sensory performance within that modality, leading to a more reliable representation of the animal’s environment^2^. This in turn should enhance behavioural performance in a way that is beneficial for the animal, thus driving natural selection^50^.

Surprisingly, the eye anatomy in these hawk moth species seems to reflect the need for visual sensitivity, rather than the importance of vision for behaviour, or the relative neural investment in vision: in absolute terms, the nocturnal *D. elpenor* has distinctly larger eyes than *M. stellatarum*, and twice the number of ommatidia^31^, and yet, proportionally, their visual neuropils are smaller than those of *M. stellatarum*. While eye size has successfully been used in ant apposition eyes as a measure for the importance of vision^8^, in hawk moths (with superposition optics) active at different light intensities, quantitative behavioural measurements as performed here, yet not eye size, provide reliable correlations with differential investment in sensory brain areas. The significantly larger eyes of the nocturnal species are used primarily to improve visual sensitivity at night rather than to increase resolution, by increasing light capture through large superposition apertures. Moreover, anatomical^31^ and physiological evidence^51^ suggests that nocturnal vision in *D. elpenor* is further enhanced by the summation of visual signals from neighbouring ommatidia, while reducing the spatial resolution. On the other hand, while the diurnal species has a smaller overall eye size and ommatidial number, their optics provide higher spatial resolution, because there is no resolution-compromising need for increased sensitivity. Thus, while the nocturnal species has a bigger eye, it invests less nervous tissue to analyze the information from each ommatidium of the eye (because the increased size serves mainly to increase sensitivity, not resolution), and this reduced investment in processing power per ommatidium is reflected in the behavioral importance of vision. Thus, eye size and ommatidial number must be used with great caution as proxies for the “importance” of vision in insects.

## Conclusions

In our study we have shown that quantitative differences in hawk moth brain morphology reflect quantitative differences in behavioural choices, thus indicating that differential investment in sensory brain areas is reflected in the behavioural relevance of these senses. This supports previous expectations for such a relationship in Lepidoptera^5^,^9^. Our results will be valuable for the interpretation of past and future quantitative anatomical studies of Lepidoptera (and insects in general), since they show that variations in the anatomy of sensory brain areas can indeed be of functional significance for behaviour.

## Experimental Procedures

### Animals

In this study, two species of hawk moths (Lepidoptera: Sphingidae, Macroglossinae) were investigated: the nocturnal elephant hawk moth *Deilephila elpenor*, and the diurnal hummingbird hawk moth *Macroglossum stellatarum.* Apart from their activity window both species share very similar lifestyles and habitats^52^, and their visual abilities have been assessed in great detail^29^,^53^,^54^,^55^,^56^. Both species can associate colour and odour cues with food sources while foraging^30^. *M. stellatarum* were cultured from wild-caught individuals collected in Spain (Mallorca) and France (Sorède). *D. elpenor* were purchased as pupae from Neil West (United Kingdom). *D. elpenor* pupae were kept for a minimum of 4 months at 5 °C to simulate winter and stimulated to eclose by transferring them to room temperature. Adults of both species were kept in flight cages, on a 14:10 day:night light regime at 26°. Animals used for histology were fed with 10% sugar solution from artificial feeders for 2–7 days prior to experiments, while animals tested in the behavioural experiments were fed with 10% sugar solution after eclosion as outlined below (see Behavioural Experiments).

### Histology

#### Synapsin Labelling

Anatomical experiments were performed on five female and five male individuals of both species. Whole-mount staining using a monoclonal anti-Synapsin antibody (obtained from Dr. Buchner, Würzburg, Germany) Cat# SYNORF1 (*Drosophila* Synapsin I isoform), RRID:AB_2315426^57^; was conducted as described in^58^. In short, brains were fixed over night at room temperature in Zinc-formaldehyde fixative (0.25% [18.4 mM] ZnCl2, 135 mM NaCl, 35 mM sucrose, 1% paraformaldehyde (PFA)^59^), washed in HEPES buffered saline (HBS) and bleached using 10% hydrogen peroxide in 0.05 M Tris-buffered saline (Tris-HCl) for four hours. Afterwards the brains were washed in Tris-HCl and treated with a fresh mixture (20:80) of dimethyl sulfoxide (DMSO): methanol for 85 min. After more washing in Tris-HCl, the brains were pre-incubated with 5% normal goat serum (NGS) in 0.01 M phosphate buffered saline with 3% TritonX-100 (PBT) over night at 4 °C. They were then incubated with 1:25 anti-Synapsin antibodies (in PBT with 1% NGS) for five days at 4 °C. After intense rinsing in PBT, a secondary antibody, goat anti-mouse conjugated to Cy5 (Cy5-GAM, 1:300; Jackson ImmunoResearch, West Grove, PA, USA; catalogue number 115–175–146) was applied for five days (in PBT with 1% NGS) at 4 °C. Successively, brains were washed intensely in PBT and in phosphate buffered saline (PBS), and dehydrated in an ethanol series of increasing concentrations (50%, 70%, 90%, 95% and 100%; 15 min each). Brains were cleared in methyl salicylate and mounted between two coverslips using Permount (Electron Microscopy Science, Hartfield, PA, USA). Plastic spacers (Zweckform No. 3510, Germany) were used to prevent squeezing of the brains.

#### Neurobiotin Injections

For neurobiotin labelling, moths were restrained by taping the thorax tightly to a holder to prevent movement of the flight muscles. The head and thorax were fixed with wax, and the head capsule was opened to expose the brain. Neurobiotin (Vector Laboratories, Burlingame, UK) crystals were applied to the tip of a borosilicate micropipette, which was inserted manually into the target brain region (the antennal lobes and the lobula complex, respectively). The brain was dissected immediately and fixed overnight at 4 °C in a fixative containing 4% paraformaldehyde, 0.25% glutaraldehyde, and 2% saturated picric acid (in 0.1 M phosphate buffer). Brains were washed 4 × 15 min in 0.1 M PBS and then incubated with Cy3-conjugated streptavidin (1:1000; Jackson ImmunoResearch, West Grove, PA, USA; catalogue number 016–160–084) for three days at 4 °C. After incubation, brains were rinsed 6 × 15 min in PBT and 2 × 20 min in PBS, dehydrated in an ascending ethanol series (50%, 70%, 90%, 95% and 100%; 15 min each), treated with a 1:1 mix of 100% ethanol and methyl salicylate for 15 min, and eventually cleared for at least 45 min in pure methyl salicylate. Finally, the brains were mounted as described for antibody labelled preparations.

### Imaging and 3D-reconstruction

Anti**-**Synapsin-labelled whole-mount preparations were imaged using a 633 nm HeNe laser on a confocal microscope (LSM 510 Meta, Zeiss, Jena, Germany) using a 10x objective (Plan Neofluar 0.45 water immersion; Zeiss). Image stacks were taken at an interval of 3 μm. The refractive index mismatch between immersion medium (RI 1.34) and the mounting medium (RI 1.52) was corrected by rescaling the images in the z-dimension by a factor of 1.14 before any further analysis. Neurobiotin-injected brains were imaged with the 561 nm DPSS laser, using a 25x objective (LD LCI Plan-Apochromat 25x/0.8 Imm Corr DIC; Zeiss) with optical sections every 1 μm.

Several image stacks were necessary to image each brain. Image stacks of the same brain were aligned to a shared coordinate frame with the software Amira (Amira 5.5.3, FEI, Hillsboro, Oregon, USA). The segmentation editor of Amira was used to create a volumetric dataset in which voxels of the image stack were assigned to individual brain areas based on anti-synapsin staining. To achieve this, selected sections in all three spatial planes were manually outlined to create a 3D neuropil-scaffold. The “wrap” function of Amira was used to eventually interpolate the complete structure of a neuropil. A polygonal surface model was finally generated to visualize the neuropils in 3D. Volumes of neuropils were calculated from the segmented neuropils using the “MaterialStatistics” function in Amira.

The lateral horn region of the brain was localized in anti-Synapsin labelled preparations by defining the area and landmarks in the neurobiotin-injected brains containing fully stained lateral horn regions as templates. We confirmed that both methods yielded similar volumes.

We obtained the proportion of visual and olfactory input to the mushroom body calices by reconstructing the projection fields in the calyx generated by olfactory projections (calyx inner zone) and visual projections (calyx outer zone, see Fig. 2E).

### Behavioural Experiments

Experiments were carried out in an experimental cage (70 × 60 × 50 cm), which was illuminated from above with 4 fluorescent tubes (Biolux OSRAM L 18 W/72 965) during the day and with 33 white LEDs during the night (providing a light intensity of 0.3 cd/m^2^ matching twilight). The cage was cleaned with ethanol before each test to prevent olfactory contamination.

Naïve moths had access to two different feeders placed 30 cm away from each other, each of which presented a combination of one colour (i.e. visual cue) and one odour (i.e. olfactory cue), which were chosen to be comparable with previous experiments on hawk moths. Artificial feeders were made using plastic syringes as the core, and blue and yellow rings as the overflow reservoirs (see Fig. 3 and^60^). Odour extracts (honeysuckle perfume oil from Interlam AB, Sweden and bergamot essential oil from Naissance, UK) were diluted in the feeding solution. The rewarded feeder was yellow and contained honeysuckle odour in 10% sugar solution, while the unrewarded feeder combined blue and bergamot odour in regular tap water. Their positions were changed randomly every day to avoid position learning^29^. Since hawk moths have an innate colour preference for blue, which can prevent learning using other sensory modalities, we trained the two species to yellow feeders, which are innately less attractive to the moths, thus insuring they learn both visual and olfactory cues (rather than following their innate preference for the blue coloured feeders)^30^.

The moths were kept in a flight cage with free access to the training feeders for up to a week. Only after they succeeded in finding and probing the correct feeder with their proboscis once in a pre-test with filled rewarded (sugar water) and unrewarded (tap water) feeders, moths entered the testing phase. The nocturnal species was tested during the first four hours of their night, whereas the diurnal species was tested during the first four hours of their day cycle. All tests were performed with empty, unrewarded feeders. Food was present *ad libitum* every day after testing, but was removed ten hours before testing. Every individual was tested once.

All moths in the testing phase were tested in one of four conditions per day (*control*: yellow feeder and honeysuckle odour vs. blue feeder and bergamot odour as in training, *only visual*: yellow vs. blue feeder, *only olfactory*: white feeders with honeysuckle vs. bergamot odour, *conflict:* yellow feeder with bergamot odour vs. blue feeder with honeysuckle odour). On a particular day, the scentless tests were always performed before tests with olfactory cues to avoid odour contamination. During a test the two feeders were simultaneously present in the cage. The animals were given three minutes to warm up their flight muscles (through shivering) and to start flying. If a moth had not started flying after 3 min it was considered as “not motivated” and removed from this particular test. The preference of the moth was tested for 5 min after it started flying. The first time a moth probed a feeder with its proboscis was counted as the animal’s choice. For each individual animal, the *control* condition was tested after all other conditions, to ensure that it had retained the learned associations until the end of the experiment.

A total of 48 *D. elpenor* individuals (21 female, 28 male) and 32 *M. stellatarum* individuals (18 female, 14 male) were tested. Not all individuals made a choice in all tests, and in general, the diurnal species was more motivated to probe at the feeders in our tests than the nocturnal species, resulting in a higher proportion of animals making a choice in both the *control* condition and the *conflict* condition (Fig. 3C).

### Data Analysis

All analyses were conducted using Matlab 2015a (The Mathworks, Natick, MA, USA) and R.

#### Brain volume

For final neuropil volumes, paired brain areas in individual brains were summed to obtain single values for each brain. The overall neuropil volume of the brain was calculated by summing the unspecified neuropil volume and the volumes of all well-defined brain regions (paired and unpaired). Averages for each species were made from five male and female brains. We then compared representative populations of both female and male moths. As neuropils did not differ significantly between males and females, both were pooled for interspecies comparison. Brain volumes were compared across species, using the non-parametric Mann-Whitney-U-test.

If not specified otherwise in the text, we compare relative neuropil volumes, normalized by the central brain neuropil volume (excluding segmented sensory neuropils) in each individual. Since the optic lobes contributed around 50% to total brain volume, they have a major influence on relative neuropil scaling. Since the optic neuropils were a part of our functional comparison of sensory neuropils, we did not normalize individual neuropils by total brain neuropil volume but by central brain neuropil volume in line with previous studies^5^,^11^. We thereby avoided inducing species-specific size distortions of central brain neuropils during normalization. To aid comparison with other studies, we included the absolute neuropil sizes, as well as the relative neuropil sizes normalized to total neuropil volume in Supplementary Table S1 as well.

In order to investigate whether differences in neuropil volume resulted from true differences in the size of the respective neuropils in the two species (grade shifts), rather than percentages differences caused by allometric scaling, we performed standardized major axis regression analysis on individual neuropils in relation to the central brain volume, using the SMATR v.3 package for R^61^,^62^. We assumed an allometric relationship of the form *y* = a* x^*b*^, which translates to the linear relationship log(y) = log(x)*b + log(a). If there was no difference in allometric scaling between species (thus in the slope b), we could test for differences in the y-axis intercept, or elevation (log(a)), called a grade shift, which indicates a true difference in neuropil volume across species (Supplementary Fig. S1, Supplementary Table S2). We estimated the extent of the shift in elevation using a grade shift index (gsi) as described by^17^: a_M_/a_D_ (Figs 1 and 2). Moreover, we tested whether the scaling relationship of a neuropil of both species combined was different from isometric scaling (the neuropil scaling at the same rate as the central brain) – provided there was no difference in allometry between species. The difference in slope from isometric scaling are expressed as the slope index (si) = b_M&D_ (Figs 1 and 2).

#### Behaviour

To compare the motivation across species, we calculated the proportion of animals extending their proboscis out of the total number of animals flying in each of the conditions. Fisher’s exact test was used for statistical comparisons. Using the same statistical test, we compared the proportion of animals extending their proboscis to the rewarded versus the unrewarded feature in the *control* condition, as well as the rewarded colour versus the rewarded odour in the *conflict* condition across species. The *visual* and *olfactory* condition did not have sufficient numbers of choices in each species to compare them statistically.

### Data accessibility

Additional data supporting this article have been uploaded as part of the electronic supplementary material.

## Additional Information

**How to cite this article**: Stöckl, A. *et al*. Differential investment in visual and olfactory brain areas reflects behavioural choices in hawk moths. *Sci. Rep.* **6**, 26041; doi: 10.1038/srep26041 (2016).

## Supplementary Material

Supplementary Information
