# Supplementary material for: Differential investment in visual and olfactory brain areas reflects behavioural choices in hawk moths
Source: Sci Rep. 2016 May 17;6:26041. doi: 10.1038/srep26041 (PMC4869021; doi:10.1038/srep26041)
Supplement: Supplementary Information [file srep26041-s1.pdf]

Differential investment in visual and olfactory brain areas reflects behavioural choices in hawk moths

Anna Stöckl[1*], Stanley Heinze[1], Alice Charalabidis[1], Basil el Jundi[1], Eric Warrant[1], Almut Kelber[1]

[1] Department of Biology, University of Lund, Sölvegatan 35, S-22362 Lund, Sweden

* correspondence to: anna.stockl@biol.lu.se

Supplementary Data

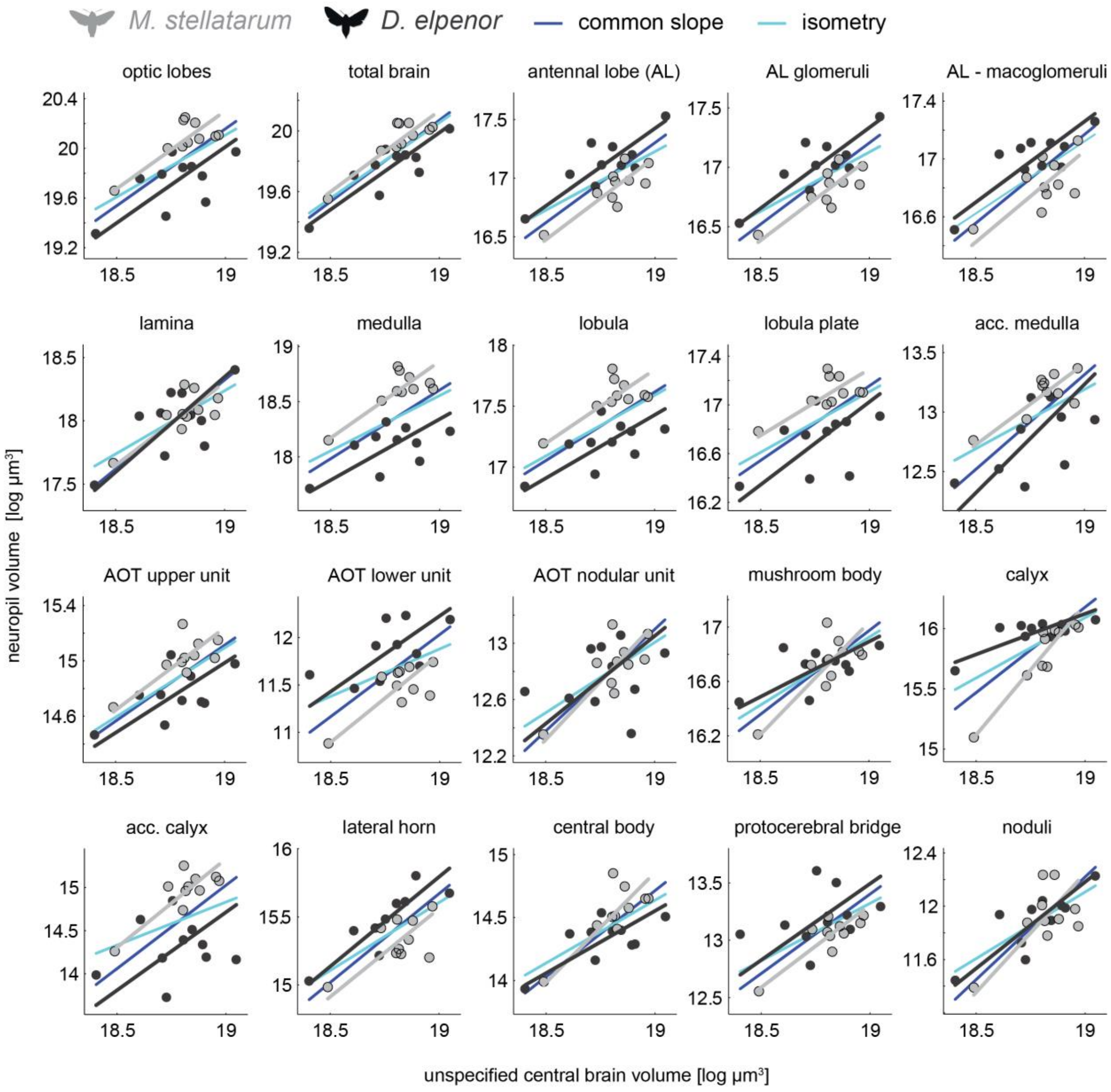

Supplementary Figure S1. Regression of neuropils with respect to central brain neuropil in *M. stellatarum* and *D. elpenor*.

Standardized major axis fits for log transformed neuropil volume in *M. stellatarum* (light grey) and *D. elpenor* (dark grey). The light blue light indicates common isometric scaling with the central brain volume, the dark blue line the common allometric slope for the two species. See also Supplementary Table S2 for full statistics.

Supplementary Table S1. Absolute and relative neuropil volume of *Macroglossum stellatarum* and *Deilephila elpenor*.

Absolute, as well as normalized neuropil (normalized by the central brain neuropil excluding the segmented neuropils, as well as by the total neuropil) of *M. stellatarum* (**M**) and *D. elpenor* (**D**). Depicted are the average (**avg**), standard deviation (**s.d.**) and standard error (**s.e.m.**) for each species (n=10). P-values (**p**) indicate significance of difference in each neuropil volume between species (Mann-Whitney-U test), and **U** values the test statistic. Yellow: p>0.05, light green: p<0.05, dark green: p<0.01.

		absolute volume [$*10^6$ μm^3]				rel. volume [% central neuropil]				rel. volume [% total neuropil]			
**structure**	**sp**	**avg**	**s.d.**	**sem**	**p & U**	**avg**	**s.d.**	**sem**	**p & U**	**avg**	**s.d.**	**sem**	**p & U**
**total brain**	M	460.583	117.647	54.409	0.009	-	-	-	-	-	-	-	-
	D	384.475	122.747	56.767	15	-	-	-	-	-	-	-	
**optic lobes**	M	263.292	76.064	35.178	0.001	-	-	-	-	57.165	16.515	7.638	<0.001
	D	189.133	72.416	33.491	7	-	-	-	-	49.193	18.835	8.711	2.000
**unspecified**	M	149.331	35.157	16.259	0.470	-	-	-	-	32.422	7.633	3.530	0.009
	D	143.741	46.515	21.512	40	-	-	-	-	37.386	12.098	5.595	15.000
**lamina**	M	70.588	11.027	5.100	0.570	47.270	7.384	3.415	0.850	15.326	2.394	1.107	0.021
	D	67.766	16.314	7.545	42	45.379	10.924	5.052	47.000	17.625	4.243	1.962	19.000
**medulla**	M	122.065	19.146	8.855	0.001	81.741	12.821	5.930	<0.001	26.502	4.157	1.922	<0.001
	D	72.831	12.578	5.817	5	48.771	8.423	3.895	0.000	18.943	3.271	1.513	0.000
**lobula**	M	43.389	6.240	2.886	0.001	29.055	4.179	1.932	0.001	9.420	1.355	0.627	<0.001
	D	29.583	4.954	2.291	6	19.810	3.317	1.534	4.000	7.694	1.288	0.596	1.000
**lobula plate**	M	26.727	3.584	1.657	0.001	17.898	2.400	1.110	0.001	5.803	0.778	0.360	0.001
	D	18.570	3.984	1.842	7	12.435	2.668	1.234	7.000	4.830	1.036	0.479	6.000
**accessory medulla**	M	0.523	0.084	0.039	0.010	0.350	0.056	0.026	0.021	0.114	0.018	0.008	0.100
	D	0.384	0.115	0.053	17	0.257	0.077	0.035	19.000	0.100	0.030	0.014	28.000
**AOT upper unit**	M	3.361	0.485	0.224	0.010	2.251	0.325	0.150	0.036	0.730	0.105	0.049	0.054
	D	2.591	0.444	0.206	16	1.735	0.298	0.138	11.000	0.674	0.116	0.053	24.000
**AOT lower unit**	M	0.099	0.020	0.009	0.010	0.067	0.014	0.006	0.002	0.022	0.004	0.002	<0.001
	D	0.147	0.039	0.018	16	0.098	0.026	0.012	8.000	0.038	0.010	0.005	0.000
**AOT nodular unit**	M	0.382	0.074	0.034	0.520	0.256	0.050	0.023	0.970	0.083	0.016	0.007	0.076
	D	0.357	0.072	0.033	41	0.239	0.048	0.022	49.000	0.093	0.019	0.009	26.000
**antennal lobe**	M	22.639	3.797	1.756	0.050	15.160	2.543	1.176	0.001	4.915	0.824	0.381	<0.001
	D	27.972	6.101	2.821	23	18.732	4.085	1.889	6.000	7.275	1.587	0.734	0.000
**lateral horn**	M	4.559	0.734	0.340	0.060	3.053	0.492	0.227	0.002	0.990	0.159	0.074	<0.001
	D	5.360	1.142	0.528	25	3.589	0.765	0.354	9.000	1.394	0.297	0.137	0.000
**mushroom body**	M	18.758	3.557	1.645	0.620	12.561	2.382	1.101	0.620	4.073	0.772	0.357	0.002
	D	18.151	2.336	1.080	43	12.155	1.564	0.723	43.000	4.721	0.608	0.281	9.000
**calyx**	M	7.492	1.699	0.786	0.076	5.017	1.138	0.526	0.011	1.627	0.369	0.171	<0.001
	D	8.656	0.880	0.407	26	5.797	0.589	0.273	16.000	2.251	0.229	0.106	0.000
**accessory calyx**	M	3.207	0.688	0.318	0.001	2.148	0.461	0.213	0.001	0.696	0.149	0.069	0.001
	D	1.697	0.522	0.241	6	1.136	0.349	0.162	5.000	0.441	0.136	0.063	6.000
**central body**	M	2.099	0.422	0.195	0.010	1.405	0.283	0.131	0.038	0.456	0.092	0.042	0.570
	D	1.687	0.259	0.120	16	1.130	0.174	0.080	22.000	0.439	0.067	0.031	42.000
**protocerebral bridge**	M	0.468	0.074	0.034	0.210	0.314	0.049	0.023	0.090	0.102	0.016	0.007	<0.001
	D	0.547	0.128	0.059	33	0.366	0.085	0.040	27.000	0.142	0.033	0.015	1.000
**noduli**	M	0.153	0.033	0.015	1.000	0.103	0.022	0.010	0.670	0.033	0.007	0.003	0.021
	D	0.148	0.030	0.014	50	0.099	0.020	0.009	44.000	0.039	0.008	0.004	19.000

Supplementary Table S2. Results of standardized major axis regression analysis

Statistical results of regression analysis of individual neuropils in the two species (n=10 for each species) with respect to central brain volume (excluding segmented neuropil), **p** value and test statistic (**teststat**): log-likelihood analysis for common slope, Wald test for common elevation, as well as log-likelihood test for isometry of the common slope. Grade shift index (**gsi**) represents the ratio of the scaling factors for each neuropil in the two species, thus indicating by which factor the two neuropils differ between species (gsi>1: neuropil is bigger in *M.* stellatarum, gsi < 1: neuropil is bigger in *D.elpenor*). The slope index (**si**) shows the common slope for each neuropil of the two species, thus indicating by which factor the allometric scaling of this neuropil with the central brain differs from isometric scaling. Yellow: p>0.05, light green: p<0.05, dark green: p<0.01.

	**common slope**		**common elevation**		**isometry**			
**structure**	**p**	**teststat**	**p**	**teststat**	**p**	**teststat**	**gsi**	**si**
optic lobes	0.907	0.014	<0.001	20.840	0.453	1.582	1.357	1.234
total brain	0.835	0.044	<0.001	19.290	0.788	0.476	1.168	1.075
antennal lobe (AL)	0.998	0.000	<0.001	21.530	0.081	5.035	0.787	1.359
AL glomeruli only	0.860	0.031	<0.001	20.380	0.079	5.069	0.786	1.369
AL - macroglomeruli	0.723	0.126	<0.001	12.350	0.723	0.126	0.812	1.203
lamina	0.551	0.355	0.790	0.071	0.156	3.721	1.020	1.385
medulla	0.649	0.208	<0.001	46.880	0.387	1.899	1.622	1.260
lobula	0.809	0.058	<0.001	29.800	0.782	0.492	1.427	1.128
lobula plate	0.479	0.502	<0.001	17.220	0.553	1.184	1.415	1.181
accessory medulla	0.351	0.872	0.004	8.358	0.071	5.286	1.349	1.493
AOT upper unit	0.781	0.077	<0.001	14.100	0.810	0.421	1.263	1.106
AOT lower unit	0.804	0.061	<0.001	16.880	0.021	7.760	0.652	1.740
AOT nodular unit	0.605	0.268	0.814	0.055	0.118	4.278	1.026	1.500
mushroom body	0.076	3.156	0.841	0.040	0.098	4.637	0.986	1.298
calyx	<0.001	12.970						
accessory calyx	0.876	0.025	<0.001	15.800	0.003	11.380	1.820	1.960
lateral horn	0.835	0.043	0.001	10.330	0.242	2.837	0.816	1.311
central body	0.116	2.465	0.016	5.796	0.052	5.903	1.184	1.429
protocerebral bridge	0.972	0.001	0.055	3.693	0.130	4.076	0.832	1.387
noduli	0.426	0.635	0.903	0.015	0.053	5.878	0.991	1.455

Supplementary Table S3. Behavioural performance of *Macroglossum stellatarum* and *Deilephila elpenor*.

Number of animals of *M. stellatarum* (**M**) and *D. elpenor* (**D**) choosing the rewarded (+) or the unrewarded (-) stimulus in the *control*, *visual*- and *olfaction*-only condition, as well as the visual (**v**) or the olfactory (**o**) stimulus in the *conflict* condition, out of the total numbers of tested animals (bold).

sp	control (+/-)	visual (+/-)	olfaction (+/-)	conflict (v/o)
M	**31** (23/2)	**32** (24/1)	**32** (0/0)	**28** (18/6)
D	**40** (18/1)	**34** (1/3)	**38** (13/0)	**34** (8/3)

Supplementary Table S4. Statistics for behavioural measurements.

Results of Fisher's exact test (**p** value and odds ratio (**OR**)) for (a) choice differences in the four test conditions (*control*, *visual*, *olfaction*, *conflict*) between *M. stellatarum* and *D. elpenor*, and differences between the proportion of animals making a foraging choice out of all tested animals for each condition vs the *control* condition in (b) *M. stellatarum* and (c) *D. elpenor*. Yellow: p>0.05, light green: p<0.05, dark green: p<0.01.

condition		control	visual	olfaction	conflict
(a) choice difference of M & D	**p**	1	-	-	0.0115
	**OR**	1.5652	-	-	0.125
(b) foraging choice / flights M	**p**		1	<0.0001	0.7342
	**OR**		0.8571	<0.0001	1.44
(c) foraging choice / flights M	**p**		1.86E-04	0.2579	0.2373
	**OR**		0.1195	0.5747	0.5286
